# Reducing demand for antibiotic prescriptions: evidence from an online survey of the general public on the interaction between preferences, beliefs and information, United Kingdom, 2015

**DOI:** 10.2807/1560-7917.ES.2018.23.25.1700424

**Published:** 2018-06-21

**Authors:** Laurence S J Roope, Sarah Tonkin-Crine, Christopher C Butler, Derrick Crook, Tim Peto, Michele Peters, A Sarah Walker, Sarah Wordsworth

**Affiliations:** 1 Health Economics Research Centre, Nuffield Department of Population Health, University of Oxford, Oxford, United Kingdom; 2 The National Institute for Health Research Health Protection Research Unit in Healthcare Associated Infections and Antimicrobial Resistance at the University of Oxford, Oxford, United Kingdom; 3 Nuffield Department of Primary Care Health Sciences, University of Oxford, Oxford, United Kingdom; 4 National Institute for Health Research Oxford Biomedical Research Centre, John Radcliffe Hospital, University of Oxford, Oxford, United Kingdom; 5 Nuffield Department of Clinical Medicine, University of Oxford, John Radcliffe Hospital, University of Oxford, Oxford, United Kingdom; 6 Oxford University Hospitals National Health Service Trust, Oxford, United Kingdom; 7 Health Services Research Unit, Nuffield Department of Population Health, University of Oxford, Oxford, United Kingdom

**Keywords:** antibiotic use, antimicrobial resistance, Time preference, Risk-aversion, Information, Survey

## Abstract

Background: Antimicrobial resistance (AMR), a major public health threat, is strongly associated with human antibiotic consumption. Influenza-like illnesses (ILI) account for substantial inappropriate antibiotic use; patient understanding and expectations probably play an important role. Aim: This study aimed to investigate what drives patient expectations of antibiotics for ILI and particularly whether AMR awareness, risk preferences (attitudes to taking risks with health) or time preferences (the extent to which people prioritise good health today over good health in the future) play a role. Methods: In 2015, a representative online panel survey of 2,064 adults in the United Kingdom was asked about antibiotic use and effectiveness for ILI. Explanatory variables in multivariable regression included AMR awareness, risk and time preferences and covariates. Results: The tendency not to prioritise immediate gain over later reward was independently strongly associated with greater awareness that antibiotics are inappropriate for ILI. Independently, believing antibiotics were effective for ILI and low AMR awareness significantly predicted reported antibiotic use. However, 272 (39%) of those with low AMR awareness said that the AMR information we provided would lead them to ask a doctor for antibiotics more often, significantly more than would do so less often, and in contrast to those with high AMR awareness (p < 0.0001). Conclusion: Information campaigns to reduce AMR may risk a paradoxical consequence of actually increasing public demand for antibiotics. Public antibiotic stewardship campaigns should be tested on a small scale before wider adoption.

## Introduction

Antimicrobial resistance (AMR) is a growing threat to global health and the economy. A recent report commissioned by the United Kingdom (UK) government estimated that AMR could reduce global gross domestic product by 2–3.5%, costing up to USD 10^14^ (EUR 1.39 × 10^14^) from 2014 to 2050 [1]. Many pathogens are becoming resistant faster than alternative antibiotics are being developed [2]. There are strong associations between the volume of human antibiotic consumption and AMR [3]. Reducing AMR requires better antibiotic stewardship [4,5].

Respiratory tract infections (RTIs) account for most human antibiotic use [6,7]. However, RTIs are often viral, and more than half of the antibiotics taken for influenza-like-illnesses (ILI) are unnecessary. Even in healthcare systems such as the UK’s National Health Service, where general practitioners (GPs) act as gatekeepers to prescriptions, patient expectations probably play an important role in unnecessary antibiotic consumption [8,9]. Several multi-faceted public campaigns in high-income countries have successfully reduced antibiotic use, but isolating the impact of campaign elements intended to improve AMR awareness is difficult [10].

A large proportion of the public in the UK believe antibiotics are effective for ILI [11]. However, little is known about the links between AMR awareness and expectations of antibiotics, or of the influence of risk preferences (attitudes to taking risks with health) or time preferences (the extent to which people prioritise good health today over good health in the future). These latter characteristics predict other health-related behaviours [12-14]; we hypothesised that they might be associated with inappropriate antibiotic use. Moreover, the probable impact of public information campaigns about AMR on antibiotic demand is uncertain. Therefore, we surveyed a representative sample of the general adult population in the UK to assess AMR awareness, the role that this and other factors (particularly risk and time preferences) play in unnecessary antibiotic consumption for ILI, and the impact of information provision (awareness campaigns) on future unnecessary antibiotic use. The study’s key outcomes were individuals’ beliefs about effectiveness and reported use of antibiotics for ILI.

## Methods

### Survey design

We developed a web-based survey to ask the general public questions about antibiotics and AMR. Survey development was informed by discussions with three clinical experts (a GP and two junior doctors) and a patient and public involvement group (n = 7).

The survey (Supplement 1) asked respondents to consider Health State A: ***“****You have a fever, aching muscles, a headache, a dry chesty cough, a sore throat, and tiredness.****”*** This description was intended to convey ILI, for which antibiotics are often expected but not generally necessary or useful. Health State A was described symptomatically, rather than specifying a ‘flu’ or ‘virus’, for two reasons: Firstly, individuals may interpret such terms differently. Secondly, people may know that antibiotics are inappropriate for viral conditions, yet not recognise Health State A symptoms as more consistent with a viral than a bacterial infection.

Respondents were asked how they would respond in the hypothetical situation of having experienced Health State A for 5 days – in particular whether they would go to a doctor and request antibiotics. They were also asked whether they thought antibiotics would be likely to help this condition, and about their antibiotic use in the previous 12 months for similar conditions. Participants with dependent children then answered analogous questions about the hypothetical situation of their youngest child being ill.

The survey captured information on a wide range of factors that might be associated with patient expectations for, and use of, antibiotics, including sociodemographic factors such as age, sex, ethnicity and health. A short-form questionnaire [15] captured personality traits – ‘extraversion’, ‘agreeableness’, ‘conscientiousness’, ‘neuroticism’ and ‘openness.’ The survey also elicited respondents’ attitudes to taking risks with health (risk preferences), and their prioritisation of good health today over good health in the future (time preferences).

The respondents were then given information (Box) that paraphrased text on the websites of four institutions aiming to improve awareness of AMR [16-19]. Participants were asked how surprising they found the information, and how it would influence (i) their future attendance at a doctor’s surgery for conditions like Health State A; (ii) their likelihood of asking a doctor for antibiotics in future. Participants with children were asked analogous questions regarding how they would act for their youngest child.

Box **Information about antimicrobial resistance given to questionnaire respondents, United Kingdom, 2015**

“*Antibiotic resistance occurs when an antibiotic loses its ability to effectively control or kill growing bacteria. It is an increasingly serious threat to public health. Without effective antibiotics, many routine treatments will become increasingly dangerous. Setting broken bones, and even basic operations, rely on access to antibiotics that work. Antibiotic resistance is believed to be caused by unnecessary use of antibiotics, and inappropriate use, such as not taking them as prescribed, skipping doses, or saving them for later use.*”

#### Risk and time preferences

Risk preferences are typically elicited in surveys via a series of questions involving the choice between a lottery and a monetary outcome, e.g. “*Which would you prefer to receive today: EUR 50, or a 50% chance of receiving nothing and a 50% chance of receiving EUR 100.*” However, recent evidence suggests that risk (and time) preferences are domain-specific [20,21]. We therefore framed our elicitation method in a health context.

Alongside Health State A respondents were asked to consider Health State B: “*You have a fever, chest pain, night sweats, a cough that brings up phlegm, loss of appetite, extreme tiredness, and some weight loss.*” Health State B was intended to represent a substantially more serious condition than A, such as tuberculosis or lung cancer. Respondents were asked to assign each health state a value from 0 (worst imaginable health state) to 10. For the method to work, we needed respondents to recognise that Health State B was worse than A. However, it was important that it did not sound so severe that few respondents would ever take a gamble in which Health State B was a possible outcome. Following an internal pilot (n = 152 participants), the words ‘…and some weight loss’ were added to the original Health State B description to encourage more respondents to evaluate Health State B as more severe than A.

Attitudes towards risk were elicited via a set of standard gamble questions with two options. The options varied in terms of the fictitious health state lasting 2 weeks that would be assigned to the respondent. In each question, Option 1 was described as being like a lottery: a respondent might be in Full Health, but could instead be in Health State B. In Option 2, the respondent would be guaranteed Health State A. Throughout the questions, the probability of Health State B in Option 1 was varied, to elicit the probability required for a respondent to be indifferent between choosing Option 1’s gamble and Option 2’s guaranteed health state. Together with the subjective health state evaluations, this enabled construction of a risk aversion variable.

Time preferences were elicited via a similar series of questions, each containing two options. In Option 1, respondents would immediately face 2 weeks of Health State A, followed by Full Health for the next 18 years. In Option 2, respondents would instead face 2 weeks of Health State B, at some specified time in the next 18 years, and Full Health at all other times. Throughout the questions, the timing of Health State B in Option 2 was varied, to elicit how far into the future it should occur for a respondent to be indifferent between Health State A now, and Health State B then. This enabled construction of variables indicating the extent to which respondents discount the future, including those who were never happy to accept Health State B, no matter how far into the future, but preferred Health State A today. The latter individuals are referred to hereafter as ‘very low discounters’. This indicates an extreme time preference where the personal discount rate is close to zero.

### Survey participants

The survey was conducted online using a panel of respondents provided by Survey Sampling International (SSI), a data collection and market research company. SSI was commissioned to obtain a sample of at least 2,000 completed responses, representative of adult members of the general public in terms of sex, age, ethnicity and geographic region. The sample size was powered to quantify the proportion of individuals who believe antibiotics are effective for ILI, at a 95% confidence level, with 2.5% precision and assuming 25% missing responses. Survey invitations were emailed to 6,280 SSI panel members resident in the UK.

A small incentive, worth approximately EUR 0.83, was offered for survey completion in the form of ‘Nectar points’ (a UK loyalty card scheme via which customers accrue discounts redeemable at various outlets). The invitations were sent over 3 weeks in May and June 2015. The first 152 responses were collected in an internal pilot, to confirm that there were no unexpected technical problems. As noted above, following the internal pilot, an amendment was made to the description of Health State B.

The SSI survey was undertaken outside the NHS setting and therefore did not need NHS ethical approval. Completion of the questionnaire was considered as indicating consent. Respondents were able to refuse to participate in the questionnaire at any stage in the process. All data were processed in accordance with the UK Data Protection Act 1998.

### Statistical analysis

Four dependent variables were initially modelled using probit regressions, each with several sets of independent variables; ‘Believe antibiotics would help Health State A’, ‘Find AMR information surprising’, ‘Would ask doctor for antibiotics if I went’, and ‘Have taken antibiotics for a condition similar to Health State A in last 12 months’. The same models were then fitted to the dependent variables ‘definitely/probably visit a doctor more often’ and ‘definitely/probably ask a doctor for antibiotics more often’ in response to the provided AMR information. There was a ‘prefer not to answer’ option for questions on income, sex, ethnicity, religion and education, leading to slightly lower sample sizes for models including these independent variables. All models used complete cases (i.e. no missing variables) for specific groups of independent variables. Model 1 included sociodemographic data. Model 2 additionally adjusted for discounting and risk aversion. Model 3 also adjusted for personality. Model 4 also adjusted for awareness of AMR and that antibiotics are unlikely to benefit ILI. Coefficients for sociodemographic and respondent characteristics in Models 2, 3 and 4 therefore correspond to residual effects not mediated through their impact on discounting, risk aversion, personality or awareness of AMR and the ineffectiveness of antibiotics for ILI. The 152 pilot participants were excluded from all models including risk aversion and time preferences. The relative quality of Models 1–4 was assessed by both the Akaike information criterion (AIC) and Bayesian information criterion (BIC). These standard tests assign values to each model and deem the model with the minimum value to have the highest quality. Subgroups of respondents sharing similar characteristics were identified using hierarchical clustering of the sociodemographic, health and personality variables. Income is reported in this paper in EUR, using the conversion rate from GBP applicable on 31 May 2015.

## Results

### Respondent characteristics

2,064 (33%) of the 6,280 individuals contacted completed the survey. An additional 223 people began the questionnaire but did not complete it. A further 102 individuals clicked on the link in the invitation email but did not begin the questionnaire.

Table 1 provides some key characteristics of the respondents, including personality traits and risk and time preference indicators. The latter variables could only be estimated in 1,214 and 1,192 respondents, respectively, who evaluated Health State B as worse than A. Elicitation of time preference variables additionally required respondents to assign Health State A a score under 10 (Full Health). Those with and without risk/time indicators were broadly similar in terms of age, income, ethnicity, employment, country of origin and region of residence. However, fewer respondents with preference indicators were male (p = 0.0006), Christian (p = 0.007) or married/partnered (p = 0.002), and more had a higher education (p = 0.003). Those with preference indicators had lower ‘extraversion,’ (p = 0.001) and more ‘neuroticism’ (p = 0.03) and ‘openness’ (p = 0.04).

**Table 1:** Respondent characteristics, behavioural survey on antibiotic prescriptions, United Kingdom, 2015 (n = 2,064)

Variable	Full sample			Restricted sample with risk and time preferences			p value^a^
	N with data	Mean	SD	N with data	Mean	SD	
Age	2,064	44	15.7	1,117	44.0	15.9	0.55
Household equivalent income (EUR)	1,886	31,066	25,487	1,027	31,672	25,373	0.26
Own self-rated health (0–10)	2,064	7.3	2.0	1,117	7.5	1.8	0.0001
	N with data	n	%	N with data	n	%	
Male	2,061	994	48.2	1,117	500	44.8	0.0006
White	2,042	1,821	89.2	1,107	1,000	90.3	0.07
Christian	2,007	1,010	50.8	1,097	527	48.0	0.007
Higher-education	2,047	954	46.6	1,112	552	49.6	0.003
Unemployed	2,064	105	5.1	1,117	56	5.0	0.87
Sick/disabled	2,064	82	4.0	1,117	40	3.6	0.32
Married/civil partnership/live with partner	2,064	1,351	65.5	1,117	697	62.4	0.002
United Kingdom-born	2,064	1,856	89.9	1,117	1,006	90.1	0.82
Geographic region							
East Anglia	2,064	171	8.3	1,117	107	9.6	0.02
East Midlands	2,064	129	6.3	1,117	77	6.9	0.19
West Midlands	2,064	181	8.8	1,117	95	8.5	0.65
London	2,064	297	14.4	1,117	160	14.3	0.93
North East	2,064	80	3.9	1,117	36	3.2	0.10
North West	2,064	239	11.6	1,117	132	11.8	0.71
South East	2,064	337	16.3	1,117	191	17.1	0.30
South West	2,064	180	8.7	1,117	92	8.2	0.40
Yorkshire and Humberside	2,064	166	8.0	1,117	76	6.8	0.02
Wales	2,064	91	4.4	1,117	49	4.4	0.96
Scotland	2,064	161	7.8	1,117	87	7.8	0.98
Northern Ireland	2,064	32	1.6	1,117	15	1.3	0.41
Personality and behaviour^b^							
	N with data	Mean	SD	N with data	Mean	SD	
Extraversion	2,064	5.9	1.9	1,117	5.8	2.0	0.001
Agreeableness	2,064	7.0	1.6	1,117	6.9	1.6	0.20
Conscientiousness	2,064	7.7	1.7	1,117	7.7	1.7	0.94
Neuroticism	2,064	5.8	2.1	1,117	5.9	2.2	0.03
Openness	2,064	6.8	1.6	1,117	6.9	1.7	0.04
Risk-averse^c^	NR	NR	NR	1,117	2.389	2.386	NR
	N with data	n	%	N with data	n	%	
Very low discounter	NR	NR	NR	1,117	337	30.2	NR

NR: not reported; SD: standard deviation.

^a^ p values from t-tests for continuous and chi-squared test for categorical factors compare those with and without risk and time preference indicators.

^b^ Extraversion, agreeableness, conscientiousness, neuroticism, and openness were measured on a scale from 2 to 10.

^c^ The risk-averse variable was measured on a scale from −9 to 9.

### Beliefs about antibiotic use

A total of 988 (48%) respondents said they would ‘definitely/probably’ visit the GP if they experienced Health State A for 5 days. In this situation, 706 of 1,816 (39%) respondents would ask a GP for antibiotics. (The denominator here is less than 2,064 because 248 respondents said they would ‘definitely not’ visit the GP if they experienced Health State A for 5 days. In the survey (Supplement 1), these respondents were not asked the subsequent question “*If you went to see a GP about this condition, do you think you would request antibiotics?*”) Some 762 (37%) believed that antibiotics would ‘definitely/probably’ help and 426 (21%) respondents reported taking antibiotics for ILI in the last 12 months. Some 430 (53%) respondents believed that antibiotics would help their child if they had ILI, while 200 (25%) said that their child had taken antibiotics for ILI during the last 12 months. A total of 705 (34%) respondents were ‘very/somewhat’ surprised by the provided AMR information.

Models of ‘Believe antibiotics would help Health State A’, ‘Find AMR information surprising’, ‘Would ask doctor for antibiotics if I went’ and ‘Have taken antibiotics for a condition similar to Health State A in last 12 months’ indicated associations with several sociodemographic characteristics (Supplement 2, Tables 2.1–2.4). Being more extraverted was significantly positively associated with these outcomes, while being more conscientious, more open and a very low discounter were negatively associated. Adjusting for these traits, effect size and significance of many demographic characteristics declined or disappeared, and generally declined further after also adjusting for AMR awareness and for knowledge that antibiotics are unlikely to help ILI.

### Reported response to AMR information

Of the 705 who found the AMR information ‘very/somewhat’ surprising, 283 (40%) said it would lead them to ‘definitely/probably’ visit a doctor more often, while 272 (39%) said it would lead them to ‘definitely/probably’ ask for antibiotics more often. These proportions were significantly higher than the respective percentages for ‘definitely/probably’ visiting a doctor, and asking for antibiotics less often (92 (13%) and 112 (16%), respectively; Wilcoxon single-sample sign-rank p < 0.0001) (Table 2). This distribution of responses differed significantly from those who did not find information on AMR surprising (both Wilcoxon rank-sum p < 0.0001). This adverse reaction to the information, among those surprised by it, was even stronger in the context of taking one’s child to the doctor and requesting antibiotics for them.

**Table 2:** How survey respondents said information about AMR would affect the number of times that they and their children visit a doctor and request antibiotics for conditions like Health State A, United Kingdom, 2015 (n = 2,064)

	For oneself (adult)				For one’s child			
	Find information on AMR surprising (n=705)		Don’t find information on AMR surprising (n=1,359)		Find information on AMR surprising (n=370)		Don’t find information on AMR surprising (n=446)	
	n	%	n	%	n	%	n	%
Visits to GP								
Definitely visit less	29	4.1	131	9.6	10	2.7	16	3.6
Probably visit less	63	8.9	142	10.5	31	8.4	48	10.8
No change	309	43.8	999	73.5	158	42.7	340	76.2
Probably visit more	194	27.5	29	2.1	100	27.0	16	3.6
Definitely visit more	89	12.6	16	1.2	63	17.0	7	1.6
Don’t know	21	3.0	42	3.1	8	2.2	19	4.3
p (change within group)^a^	< 0.0001		< 0.0001		< 0.0001		< 0.0001	
p (difference between groups)^b^	< 0.0001				< 0.0001			
Ask for antibiotics								
Definitely ask less	40	5.7	194	14.3	18	4.9	31	7.0
Probably ask less	72	10.2	182	13.4	48	13.0	63	14.1
No change	303	43.0	897	66.0	132	35.7	312	70.0
Probably ask more	179	25.4	23	1.7	105	28.4	13	2.9
Definitely ask more	93	13.2	8	0.6	54	14.6	6	1.4
Don’t know	18	2.6	55	4.1	13	3.5	21	4.7
p (change within group)^a^	< 0.0001		< 0.0001		< 0.0001		< 0.0001	
p (difference between groups)^b^	< 0.0001				< 0.0001			

AMR: antimicrobial resistance; GP: general practitioner; ILI: influenza-like-illness

^a^ Wilcoxon single-sample signed-rank test that the median value within each group is no change.

^b^ Wilcoxon rank-sum test comparing the distribution of responses in those who do and do not find the AMR information surprising.

p values calculated excluding ‘Don’t know’ category and coding a 5-point Likert scale for each variable.

Of the 1,359 who reported finding the AMR-information ‘not very/not at all’ surprising, 273 (20%) said it would lead them to ‘definitely/probably’ visit a doctor less often, while 376 (28%) said it would lead them to ‘definitely/probably’ ask for antibiotics less often, significantly higher than the respective percentages for ‘definitely/probably’ visiting a doctor, and asking for antibiotics, more often (Wilcoxon single-sample sign-rank p < 0.0001). The majority of those who found the AMR information ‘not very/not at all’ surprising said it would lead to ‘no change’ in how often they would visit a doctor (n = 999; 74%) or ask for antibiotics (n = 897; 66%).

Several factors were univariably associated with ‘definitely/probably’ asking for antibiotics more often (Table 3). 

**Table 3:** Differences in characteristics of those who would and would not ‘definitely/probably’ ask a doctor for antibiotics more often after receiving information about AMR, United Kingdom, 2015 (n = 1,991)

	Would ‘definitely/probably’ ask doctor for antibiotics more often			‘No change’ or would ‘definitely/probably’ ask doctor for antibiotics less often			Univariable^a^	Multivariable^b^
Sociodemographic data								
	N with data	n	%	N with data	n	%	p value	p value
Male	302	174	57.6	1,687	781	46.3	0.0003	0.04
White	300	233	77.7	1,674	1532	91.5	< 0.0001	0.60
Christian	293	143	48.8	1,645	848	51.6	0.39	0.50
Higher education	301	156	51.8	1,676	769	45.9	0.06	0.74
Unemployed	303	13	4.3	1,688	84	5.0	0.61	0.74
Sick/disabled	303	11	3.6	1,688	69	4.1	0.71	0.11
Married/partnered	303	221	72.9	1,688	1089	64.5	0.004	0.03
United Kingdom-born	303	262	86.5	1,688	1,535	90.9	0.02	0.96
	N with data	Mean	SD	N with data	Mean	SD	p value	p value
Age (years)	303	35	11.6	1,688	46	15.8	< 0.0001	0.47
Household income	287	EUR 30,793	EUR 26,049	1,539	EUR 31,226	EUR 25,492	0.80	0.30
Own self-rated health (0–10)	303	7.0	2.0	1,688	7.4	2.0	0.0009	0.08
Personality								
Extraversion	303	6.0	1.4	1,688	5.9	2.0	0.23	0.26
Agreeableness	303	6.9	1.5	1,688	7.0	1.6	0.53	0.82
Conscientiousness	303	7.1	1.7	1,688	7.8	1.7	< 0.0001	0.27
Neuroticism	303	5.8	1.7	1,688	5.8	2.2	0.92	0.85
Openness	303	6.4	1.4	1,688	6.9	1.7	< 0.0001	0.002
Risk and time preferences								
Risk aversion	130	1.6	2.5	1,027	2.5	2.4	0.0003	0.11
	N with data	n	%	N with data	n	%	p value	p value
Very low discounter	139	7	5.0	1,038	342	32.9	< 0.0001	< 0.0001
Attitudes to antibiotics and ILI								
Surprised by AMR information	303	272	89.8	1,688	415	24.6	< 0.0001	< 0.0001
Believe antibiotics would help Health State A	303	232	76.6	1,688	514	30.5	< 0.0001	< 0.0001
Taken antibiotics for Health State A in last 12 months	303	169	55.8	1,688	245	14.5	< 0.0001	ND
	N with data	Mean	SD	N with data	Mean	SD	p value	p value
Health State A rating	303	6.3	2.3	1,688	5.5	2.1	< 0.0001	ND

AMR: antimicrobial resistance; ILI: influenza-like illness; ND: not done; SD: standard deviation.

^a^ t-test for continuous and chi-squared test for categorical factors.

^b^ From a multivariable probit model including all factors. Model coefficients and further details are available in Supplement 2, Table 2.5, final column.

In all specifications of the multivariable models (Figure), those who would ‘definitely/probably’ visit and ‘definitely/probably’ ask a doctor for antibiotics more often were significantly more likely to be male and married/partnered. They were independently significantly more likely to believe antibiotics are likely to help ILI, and more likely to be surprised by our AMR information, but less likely to be a very low discounter, and less open. Of the models including risk and time preferences, Model 4, which also adjusted for personality, AMR awareness and knowledge that antibiotics are unlikely to help ILI, was preferred to Models 2 and 3 according to both the AIC and BIC model selection tests.

**Figure:**
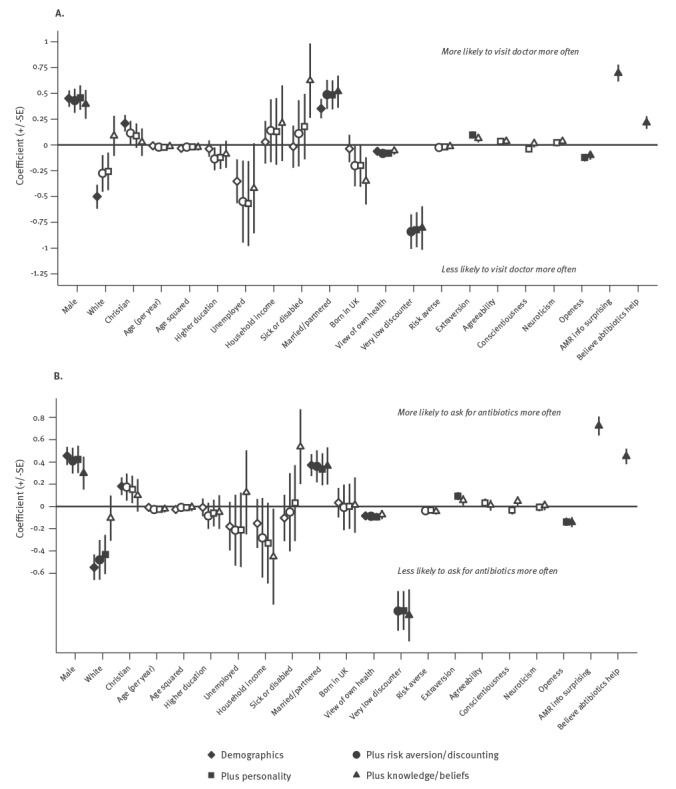
Probit regression models for the survey responses ‘visiting doctor more’ and ‘asking for more antibiotics’ in response to AMR information, United Kingdom, 2015 (n = 1,769)

AMR: antimicrobial resistance; SE: standard error; UK: United Kingdom.

Solid symbols: p < 0.05; empty symbols: p > 0.05. See Supplement 2, Table 2.5 for full model details.

### Cluster analysis

Cluster Group 1 contained most (176/276; 64%) respondents who would ‘definitely/probably’ ask for antibiotics more often, while Group 5 contained most of the remainder (66/276; 24%) (Table 4). Group 5 contained the highest percentage of people who would ‘definitely/probably’ ask for antibiotics more often (66/264; 25%), and Group 1 the second highest (176/1,055; 17%). Group 1 and Group 5 had more income and were younger than the survey sample averages. Group 1 was 99% (1,046/1,055) white, almost 100% (1,050/1,055) UK-born and 48% (511/1,055) male, while 48% (511/1,055) had a higher-education. Group 5 was 33% white (88/264), 38% (101/264) UK-born and 35% (92/264) male and 72% (191/264) with a higher education.

**Table 4:** Percentage of different profiled groups ‘definitely/probably’ asking a doctor for antibiotics more often after receiving information about AMR, United Kingdom, 2015 (n = 1,816)

Response to AMR information	Cluster groups					Total
	Group 1	Group 2	Group 3	Group 4	Group 5	
Total in cluster group	1,055	77	73	347	264	1,816
‘Definitely/probably ask less often’ or ‘no change’	853	63	64	320	187	1,487
‘Definitely/probably ask more often’	176	8	9	17	66	276
‘Don’t know’	26	6	0	10	11	53
Percentage who would ‘definitely/probably ask more often’	16.7%	10.4%	12.3%	4.9%	25.0%	15.2%
Mean age (years)	*39.6*	37.1	47.8	*64.0*	*36.6*	44.0
Percentage male	48.4%	55.8%	43.8%	*60.8%*	*34.9%*	48.2%
Percentage white	99.1%	96.1%	97.3%	99.4%	*33.3%*	89.2%
Percentage United Kingdom-born	99.5%	96.1%	100.0%	100.0%	*38.3%*	89.9%
Percentage with higher education	48.4%	29.9%	*21.9%*	32.3%	*72.4%*	46.6%
Percentage sick/disabled	0%	0%	100%	0%	0%	4.0%
Mean household income (EUR)	*33,947*	12,872	15,891	29,478	*31,804*	31,066

AMR: antimicrobial resistance.

The Table shows the subset of characteristics which varied most across the different groups (key differences in italics). All characteristics are listed in Supplement 3, Table 3.1

## Discussion

In this study, lack of AMR-awareness and the belief that antibiotics would help ILI were strongly associated with self-reported likelihood of consulting and requesting antibiotics for ILI, and with antibiotic consumption. This lack of knowledge was a mediating variable in the association between consulting/requesting antibiotics for ILI and sociodemographic characteristics. This may indicate a role for public information campaigns, perhaps targeted at specific population groups. It is encouraging that respondents who were unsurprised by the AMR information provided said it would make them less likely to consult a GP or request antibiotics. It is therefore possible that over a longer timeframe, repeated exposure to AMR information could reduce antibiotic requests. However, many with poor AMR awareness said they would react to such information by consulting doctors and asking for antibiotics more, not less often. This identifies a potential paradox where the overall impact of a public campaign could be increased demand for antibiotics. Such phenomena have been found in public health campaigns outside the field of AMR [22,23]. A meta-analysis of fear-appeal health campaigns found that fear-appeal messages, while often effective, are likely to backfire if people do not believe they are able to adequately protect themselves from the threat, a possibility which seems plausible here [24].

Being a very low discounter was strongly associated with awareness of AMR and the likely minimal benefit from antibiotics for ILI, and with not consulting a doctor or requesting antibiotics for ILI. Like personality, it was a mediating variable in the association between consulting/requesting antibiotics for ILI and sociodemographic characteristics. Attaching substantial importance to health in the very distant future may encourage both the acquisition of health literacy, and avoiding choices that could damage long-term health prospects for minor short-term benefits. We found no evidence of a significant association of risk-aversion with attitudes towards antibiotic use or resistance. It cannot safely be concluded, however, that risk preferences do not play a role in the demand for antibiotics. The lack of significance could stem from risk aversion leading some people to take antibiotics, e.g. to avoid uncertainty over complications, but others to avoid antibiotics, for fear of possible AMR-related problems.

### Strengths and limitations

An important limitation is that respondents’ reported future behaviour in response to the AMR information may differ from what they would actually do in real life. The reported paradoxical behaviour, where some respondents said they would ask for antibiotics more, not less often in response to the AMR information, may not reflect their actual behaviour.

Another limitation is that only members of the online survey panel participated. Thus the sample was limited to those with internet-access, basic computer literacy and an interest in completing surveys. However, use of this panel also meant that age, sex, ethnicity, geographic region and proportion unemployed were broadly representative of the general population in the UK, although the percentage with higher education (47%) was higher than the population average (27%) and mean income was lower. 

A major strength was our inclusion of variables not generally used in epidemiological studies, relating to personality and, particularly, risk and time preferences. Knowledge of how these characteristics are associated with AMR awareness and appropriate antibiotic use could help inform public campaigns. For example, advertising budgets could be used more efficiently by concentrating advertisements in specific media (television channels/programmes, websites and social media) that are typically consumed by audiences with these characteristics. Unfortunately, the risk and time variables could only be estimated in, respectively, 58% and 59% of the sample.

### Comparison with existing literature

There have been several surveys of public perceptions of AMR and appropriate use of antibiotics in primary care and a systematic review of the public’s knowledge and beliefs about AMR [11,25]. However, none of these studies have explicitly evaluated responses to information about AMR or considered a role for risk or time preferences. Our descriptive results broadly concur with previous studies in confirming wide-ranging misunderstanding regarding antibiotic efficacy for ILI and lack of knowledge about AMR.

Several public campaigns in high-income countries have successfully reduced antibiotic use in primary care [10,26]. In France, following a multi-faceted ‘antibiotics are not automatic’ campaign, there was a substantial decrease in antibiotic prescriptions between 2002 and 2007 compared with the pre-intervention period 2000 to 2002 [27]. A review by Huttner et al. concluded that because nearly all successful campaigns were multi-faceted, it was difficult to unpick whether improvements were due to changes in the behaviour of patients, physicians or both, or whether the observed improvements were related to confounding factors which would have happened in the absence of the campaigns [10]. All campaigns reviewed tried to convey the seriousness of AMR, sometimes using a fear message; most campaigns also tried to educate the public that antibiotics are ineffective for respiratory infections. Some campaigns encouraged people to complete antibiotic courses, an area of controversy [28]. A 1999 campaign in the UK to educate the public about appropriate antibiotic use and to raise AMR awareness was associated with improved knowledge about antibiotics but there was no evidence of reduced antibiotic use [29]. Campaigns in Greece and Spain also failed to show such an effect [10].

## Conclusion

Although based on hypothetical reported future behaviour, the results of this study suggest that public information campaigns to reduce unnecessary antibiotic use may risk a paradoxical consequence of increased, rather than decreased, public demand for antibiotics. This does not negate the potentially important role such campaigns may have; several public information campaigns have proven effective as part of multi-faceted interventions to reduce unnecessary prescribing. However, it underscores the importance of testing public antibiotic stewardship information campaigns on a small scale before rolling them out widely. Choosing the right words may be critical to success, and different strategies may be needed for different population subgroups [30].

Most effective campaigns have tried to convey that AMR is a serious problem, while also explaining that antibiotics are ineffective for respiratory infections [10]. The information tested in this study contained only the former element. Information containing both elements might have performed better. One of the strongest predictors of self-reported inappropriate antibiotic use, and of negative response to the AMR information, was belief in the efficacy of antibiotics for ILI. Information explicitly challenging this belief, perhaps coupled with reassurance that cold and influenza symptoms are easily treated with rest, fluids and paracetamol, could potentially form the basis for a successful public campaign. More research is urgently needed on finding an effective way to communicate this important message.
